# Iodine deficiency in the first pregnancy trimester and intelligence in adolescence

**DOI:** 10.1007/s00394-026-03955-3

**Published:** 2026-03-31

**Authors:** Sarai M. Keestra, Marsh Königs, Nienke van Welie, Kim Dreyer, Jaap Oosterlaan, Velja Mijatovic, Tessa J. Roseboom, Martijn J. J. Finken

**Affiliations:** 1 Department of Paediatric Endocrinology, Emma Children’s Hospital, Amsterdam UMC, Vrije Universiteit Amsterdam, Amsterdam, The Netherlands; 2 Department of Epidemiology and Data Science, Amsterdam UMC, University of Amsterdam, Amsterdam, The Netherlands; 3 Amsterdam Reproduction & Development Research Institute, Amsterdam UMC, University of Amsterdam, Amsterdam, The Netherlands; 4 Emma Neuroscience Group and Follow-Me Program, Department of Paediatrics, Emma Children’s Hospital, Amsterdam UMC, University of Amsterdam, Amsterdam, The Netherlands; 5 Department of Reproductive Medicine, Amsterdam UMC, Vrije Universiteit Amsterdam, Amsterdam, The Netherlands

**Keywords:** Iodine deficiency, Pregnancy, Intelligence, Neurodevelopment, ALSPAC

## Abstract

**Purpose:**

Although iodine deficiency during pregnancy has been linked to poorer childhood neurodevelopment, it is unclear whether these associations persist into adolescence. We examined whether first trimester iodine is associated with children's intelligence at age 15.

**Methods:**

We used data from 1,211 mother–child pairs in the Avon Longitudinal Study of Parents and Children (ALSPAC), with first-trimester urinary iodine-to-creatinine ratios during pregnancy and intelligence quotient (IQ) scores of the child at age 15, as assessed from the Two-Subtest Wechsler Abbreviated Scale of Intelligence. Associations were tested using linear regression adjusted for confounders. Iodine status was analysed both continuously and categorically using cut-offs (< 50, 50–99, 100–149, 150–249, > 250 µg/g). We tested interactions with child sex and maternal thyroid function.

**Results:**

Lower iodine was associated with lower Vocabulary T-scores (β = 0.72; 95% CI: 0.09–1.35), but not Matrix Reasoning or Full-Scale IQ. Children of mothers with severe iodine deficiency (< 50 µg/g) had 4.25 points lower Vocabulary T-scores (Cohen’s d = –0.40) and 3.3 points lower Full-Scale IQ (Cohen’s d = –0.27) than those with sufficient iodine status (150–250 µg/g). The associations did not differ by sex. Iodine status in the first trimester was not correlated with maternal thyroid function. However, in iodine-deficient pregnancies (< 150 µg/g), higher maternal thyroid-stimulating hormone (TSH) was associated with higher Vocabulary and Full-Scale IQ scores, a pattern not seen in iodine-sufficient pregnancies.

**Conclusion:**

Iodine deficiency in early pregnancy is associated with lower adolescent verbal intelligence and, in severe iodine deficiency, also with reduced full-scale intelligence. The observed association between higher maternal TSH and higher verbal and full-scale intelligence in iodine-deficient pregnancies suggests a possible compensatory role of the maternal thyroid axis. Adequate periconceptional iodine intake remains important and merits further study.

**Supplementary Information:**

The online version contains supplementary material available at 10.1007/s00394-026-03955-3.

## Introduction

As plant-based diets gain momentum for their planetary health benefits, there are growing concerns about their typically lower iodine contents [1–5]. As a trace element essential for thyroid hormone synthesis, iodine is especially important during early pregnancy when the demand for thyroid hormones surges to support neurodevelopment of the offspring [6–8]. Yet the foetal thyroid gland only gains its function in the second trimester, making the developing embryo dependent on maternal iodine status and thyroid hormone supply [9, 10]. Maternal iodine deficiency during gestation can lead to thyroid hormone inadequacy, which in turn may adversely impact intellectual impairment due to disruptions during critical periods of embryonic brain development [11–13]. Indeed, severe iodine deficiency has historically been linked to endemic goitre and cretinism, a condition characterised by permanent neurodevelopmental impairment, in addition to short stature and deafness [11, 14]. While the introduction of iodised salt and dietary diversification in the twentieth century significantly reduced the global prevalence of iodine deficiency disorders such as cretinism [15, 16], inadequate gestational iodine status is becoming increasingly more common in recent years [17]. Dietary shifts away from animal products naturally high in iodine such as meat, fish, shellfish, dairy, and eggs, towards plant-based alternatives less rich in this essential micronutrient may be driving this trend, which particularly affects women who are pregnant or lactating, as they have increased iodine requirements due to upregulated thyroid demands [18–20]. Another factor is the reduced consumption of salt fortified in iodine, which is driven in part by public health awareness campaigns addressing salt intake as a modifiable risk factor in cardiovascular health [21].

Unlike most European countries, the United Kingdom does not have a legislative framework mandating iodine fortification of salt [22]. A systematic review of all available dietary intake information across Europe showed that the median iodine intake of British women is below the World Health Organization's recommended daily intake of 150 µg per day [19, 23]. High rates of goitre, the tell-tale sign of iodine deficiency, was already reported among British school-aged children in the early twentieth century, particularly in the “goitre belt,” a region extending from Devon and Somerset through the Midlands to parts of northern England and southern Scotland [24–26]. A survey of 375,000 children conducted in the 1920s found goitre to be more prevalent in rural than urban areas, and up to five times more common in girls than in boys, prompting recommendations for iodine prophylaxis in endemic areas [25]. Despite the Medical Research Council recommending nation-wide iodine fortification of salt in the 1940s, no legislative action was taken at the time [4, 13]. However, iodine intake increased significantly from the 1950s to the 1980s due to general improvements in diet, but also increased milk consumption [13]. The use of iodophor disinfectants and iodine fortified cattle feed in the dairy industry alongside public health campaigns promoting milk consumption, lead to a threefold rise in iodine intake in the British population, a reduction in goitre prevalence, and the assumption of national iodine sufficiency [13]. However, more recent declines in dairy consumption and a growing preference for plant-based milk alternatives, which are often inadequately fortified with iodine, have reignited concerns about iodine deficiency in the UK [5, 19, 27]. Similar trends have been observed across Europe, where decreased dairy consumption and reduced iodised salt use contribute to re-emerging iodine deficiency [28, 29].

The United Kingdom provides a unique case study for understanding the long-term impact of iodine deficiency, particularly in pregnant women and their children, and offers critical insights on the consequences of failing to implement adequate national iodine fortification measures. A cross-sectional survey of 14–15-year-old UK schoolgirls in the early 2010s revealed widespread iodine deficiency (median urinary iodine 80.1 μg/L, below the 150 μg/L threshold which is considered adequate in women of reproductive age), with variation associated with seasonal, dietary, and geographical factors [30, 31]. This raised concerns about potential consequences on the neurodevelopment of children born to women who were iodine-deficient during pregnancy [13]. Using data from 912 mothers and children in the ALSPAC cohort, based in southwest England in the former “goitre belt,” Bath et al. (2013) [32] demonstrated that maternal iodine deficiency during pregnancy was associated with suboptimal verbal intelligence outcomes as assessed using an abbreviated form of the WISC-III in children at age eight and lower reading abilities at age nine. Children of mothers with iodine-to-creatinine ratios < 150 μg/g had higher odds of scoring in the lowest quartile for verbal IQ (OR = 1.58), reading accuracy (OR = 1.69), and reading comprehension (OR = 1.54), with performance decreasing progressively with increasing degrees of iodine deficiency. These associations were confirmed to be independent of other neurodevelopmentally important nutrient deficiencies in pregnancy, such as iron or omega-3 fatty acids, and were not accounted for by socioeconomic and psychosocial factors before or after pregnancy. A meta-analysis of individual participant data, combining 5,864 mother–child pairs from the UK, and Spain, further confirmed that maternal iodine deficiency, particularly before 14 weeks of gestation, was related to lower verbal IQ, but not performance IQ, in children between the ages of 1.5 and 8 years [33]. Verbal IQ scores were on average 5 points lower in a curvilinear pattern when maternal iodine-to-creatinine ratios were < 150 μg/g during the first trimester, with the strongest associations observed before 12 weeks [33]. However, it has not been investigated before whether the association between iodine deficiency with intelligence in the verbal domain persist in adolescence.

This study aims to evaluate whether maternal iodine deficiency during early pregnancy is associated with intelligence outcomes at age 15 years in the ALSPAC cohort, and whether maternal thyroid function modifies this association. By extending earlier findings in childhood to adolescence, we assess whether the effects of early iodine deficiency on intelligence persist and explore the role of maternal thyroid hormones. Investigating whether the effects of early gestational iodine deficiency persist or diminish as children age is important for informing public health strategies and policies aimed at reducing the long-term consequences of iodine deficiency on a population level.

## Methods

### Study design and selection of participants

**Fig. 1:**
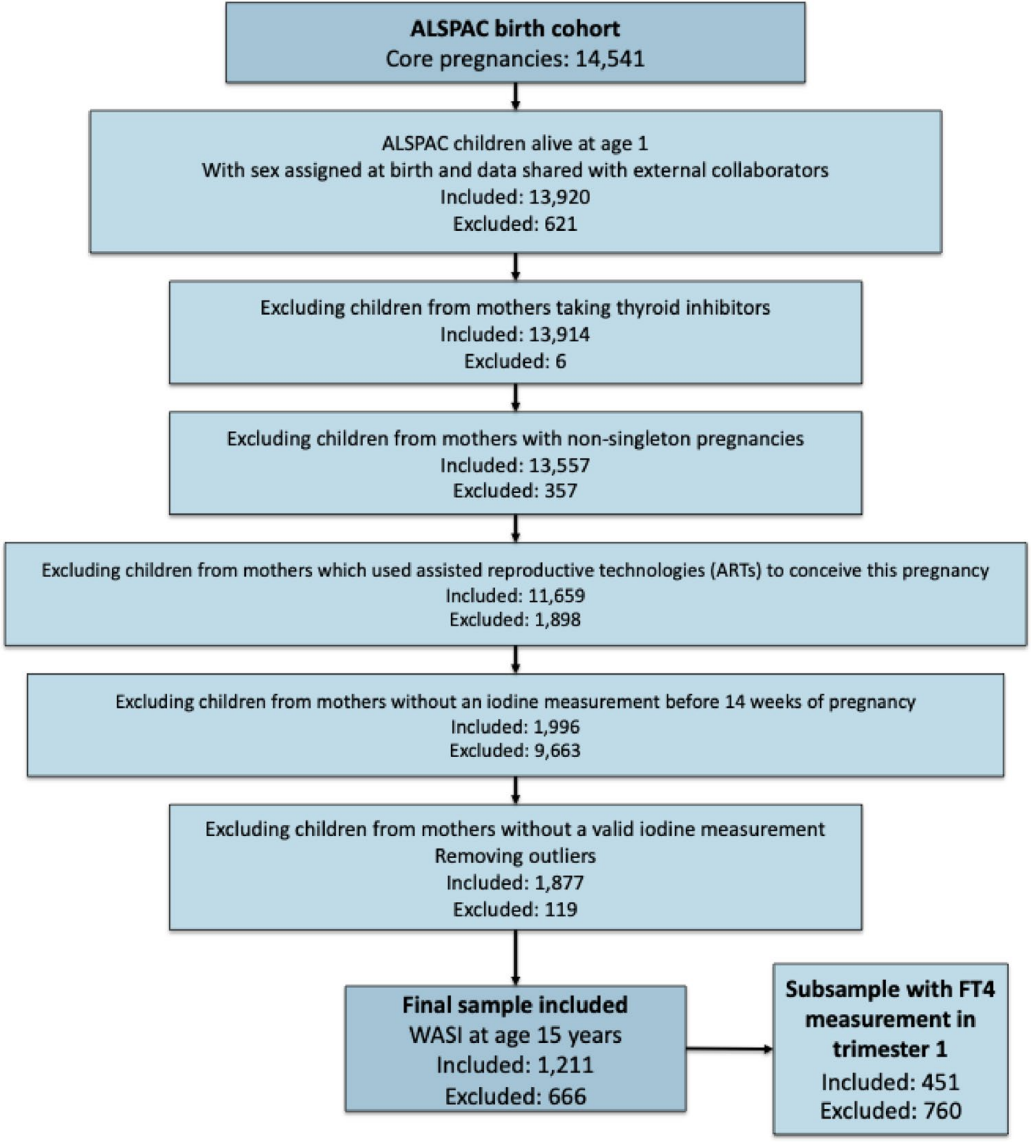
Selection of mother–child dyads from the ALSPAC cohort with valid iodine measurements and intelligence quotient available

The Avon Longitudinal Study of Parents and Children (ALSPAC) is a UK birth cohort designed to examine the health and development of children, and is extensively described elsewhere [34, 35]. In short, pregnant women resident in Avon, UK, with expected delivery dates between 1 April 1991 and 31 December 1992 met the inclusion criteria. Overall, 20,248 pregnancies were identified as being eligible and the initial number of pregnancies enrolled was 14,541. Of the initial pregnancies, there was a total of 14,676 foetuses, resulting in 14,062 live births, and 13,988 children alive at 1 year of age. These children were then longitudinally followed via questionnaires and clinic visits. Ethical approval for the study was obtained from the ALSPAC Ethics and Law Committee and the Local Research Ethics Committees. Informed consent for the use of all data collected was obtained from participants following the recommendations of the ALSPAC Ethics and Law Committee at the time. Participants can contact the study team at any time to retrospectively withdraw consent for their data to be used. Study participation is voluntary and during all data collection sweeps, information was provided on the intended use of data. The Medical Research Ethics Committee of Amsterdam UMC approved reuse of collected ALSPAC data for the purpose of the current research project. Please note that the ALSPAC study website contains details of all the data that are available through a fully searchable data dictionary and variable search tool: http://www.bristol.ac.uk/alspac/researchers/our-data/. We initially included all pregnancies from the core sample, in which children were assigned male or female sex at birth, were alive at age 1 year and whose data were shared with Amsterdam UMC as external collaborator (n = 13,920). To align our methodology with the earlier study of Levie et al. (2019) [33], we excluded pregnancies in which mothers were taking thyroid inhibitors (n = 6), carried non-singleton pregnancies (n = 357), or made use of assisted reproductive technologies (ARTs) for this pregnancy (n = 1,898) (Fig. 1), as prior use of iodinated contrast agents during fertility workups (e.g. hysterosalpingography) may affect maternal iodine status and thyroid function [36]. Based on the previous finding that iodine status in the first 14 weeks of pregnancy is most relevant for children's intelligence outcomes [33], we only included mother–child pairs in whom maternal iodine status was measured before 14 weeks of pregnancy which is when some of the most important thyroid hormone-sensitive neurodevelopment takes place [12, 37]. As concerns have been raised of potential iodine contamination by test-strips within the ALSPAC cohort [32, 33, 38], we excluded outliers above the 97th percentile of the natural-logarithmic curve for iodine after adjustment for creatinine (cut-off ≥ 743.04 µg/g). A valid urinary iodine measurement in the first trimester was available for 1,211 children that also had intelligence outcomes measured.

### Iodine status and thyroid function in the first trimester

We made use of available urinary iodine excretion data collected in mothers before 14 weeks of pregnancy. Urinary iodine excretion over 24 hours is the WHO's gold standard for assessing iodine status in individuals and populations [39]. Spot-urine samples (stored at –20 °C) were used, and iodine concentrations were adjusted for urinary creatinine to account for variation in hydration [32]. Creatinine-adjusted urinary iodine excretion is a ratio that is a widely used proxy for 24-h iodine excretion in epidemiological studies and provides a reliable estimate of iodine status [32, 39–41]. Iodine was measured at the Trace Element Unit, Southampton General Hospital United Kingdom with inductively-coupled plasma mass spectrometry (ICP-MS; SCIEX Perkin-Elmer, Beaconsfield, UK) and [42] urinary creatinine concentration was determined by the Jaffe rate method as described in earlier publications [32, 33]. As urinary iodine is not normally distributed, we log-transformed the variable before analysis and corrected for gestational week of testing in all analyses involving maternal iodine status as a continuous exposure. According to the WHO, urinary iodine excretion of < 150 µg/L is considered insufficient, whereas an excretion of 150–249 µg/L and > 250 µg/L are considered adequate and above requirements respectively [39]. These were used as the cut-offs in categorical analyses, alongside a categorisation of iodine deficiency as 100–150 µg/L as mildly deficient, 50–100 µg/L as moderately deficient, and < 50 as severely deficient in accordance to the earlier study by Bath and colleagues [32].

As a secondary exposure we made use of first trimester maternal thyroid function assessed using stored serum samples analysed on the Abbott Architect i2000 immunoassay, with a functional sensitivity of ≤ 0.05 mIU/L and intra-assay coefficients of variation < 5% and a limit detection of below 0.005 mlU/I for thyroid stimulating hormone (TSH) and of 0.3 pmol/l for free thyroxine (FT4), as described in more detail elsewhere [40–42]. The 95% range of TSH levels was 0.077–3.24mlU/I (2.5–97.5th centile), and the 95% range of FT4 levels was 12.43–22.52 pmol/l (0.97–1.75 ng/dl). Clinical ranges of TSH and FT4 during pregnancy were defined using the 2.5th–97.5th cohort-specific reference intervals as determined by Osinga et al. [43] (TSH 0.07–2.53 mU/L and FT4 12.7–22.6 pmol/L).

### Children's intelligence in adolescence

We utilised child Intelligence Quotient (IQ) data from the ALSPAC cohort to measure intelligence. At 15 years, 5,530 children completed the Two-Subtest Wechsler Abbreviated Scale of Intelligence (WASI-II), which is based on Matrix Reasoning T-scores (a subtest of performance IQ) and Vocabulary T-scores (a subtest of verbal IQ), and estimated Full-Scale IQ (FSIQ-2). The WASI-II is designed for efficient cognitive assessment, for example in a research context when administration of full test batteries is not possible [44–46]. The Vocabulary T-scores assess verbal concept formation, crystallised intelligence, and language development, while the Matrix Reasoning T-scores evaluate visual intelligence, spatial ability, inductive reasoning, and perceptual organisation [44, 47]. The two-subtest Full-Scale IQ has a mean of 100 and SD of 15 similarly to the WISC, while the subtests Matrix Reasoning and Vocabulary are expressed in T-scores with a mean of 50 and SD of 10, all age-adjusted [45]. The WASI-II has high reliability coefficients (Vocabulary: 0.94, Matrix Reasoning: 0.92, FSIQ: 0.93 in children; Vocabulary: 0.95, Matrix Reasoning: 0.94, FSIQ: 0.94 in adults) and excellent interrater agreement [44, 45]. Concurrent validity with the WISC-IV and WAIS-IV is reported as acceptable to excellent (r = 0.71–0.92), supporting its use in estimating general intelligence for research purposes [44]; detailed subtest-level correlations (e.g., between Vocabulary T-scores and verbal IQ) are reported in the technical manual. Although these subtests do not yield separate Verbal or Performance IQ scores but are instead expressed as subtest T-scores (M = 50, SD = 10), Vocabulary and Matrix Reasoning are considered domain-relevant indicators of verbal and performance intelligence-related abilities for research use, not clinical use [48–50].

### Confounders

**Fig. 2:**
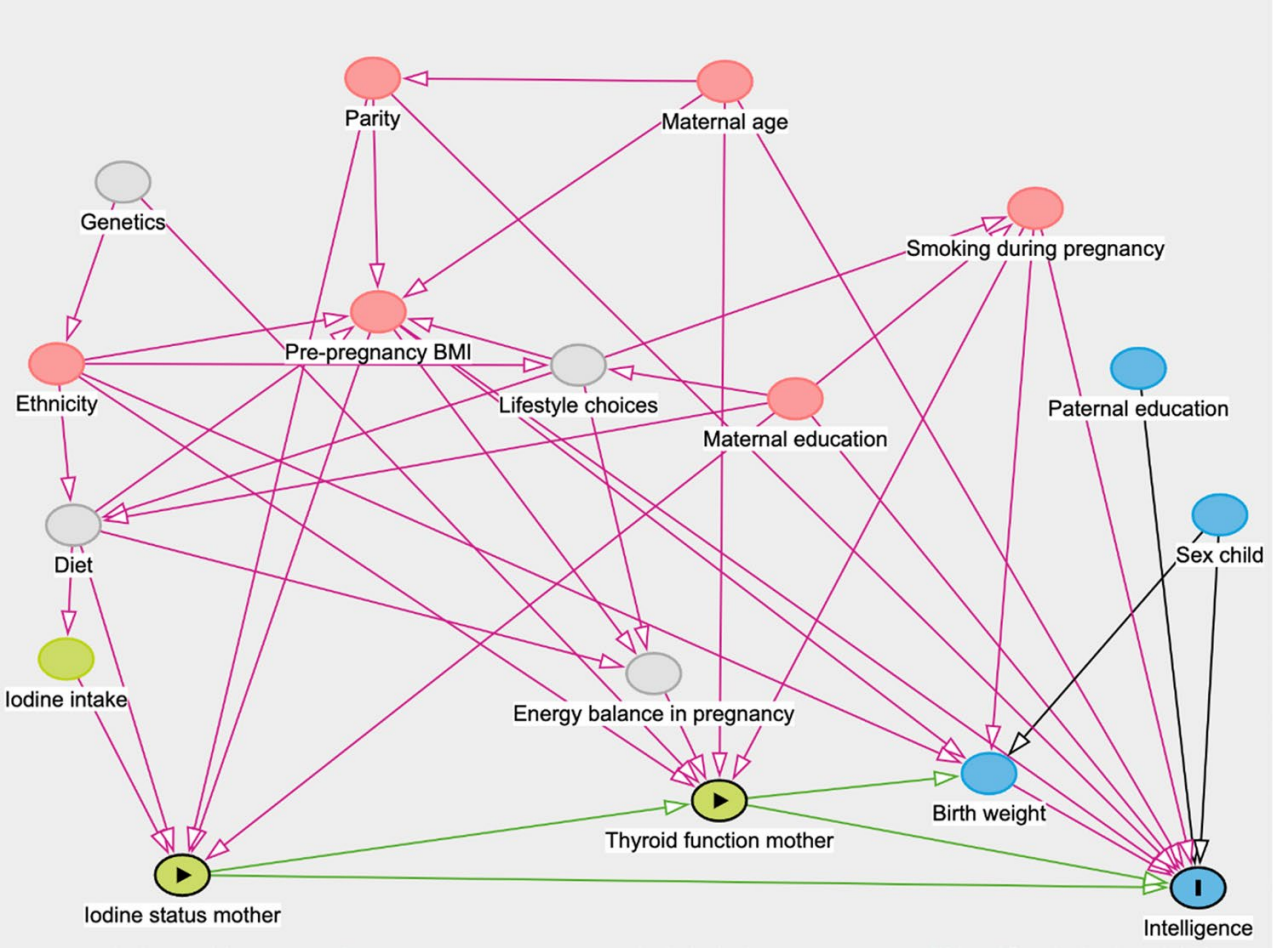
A DAG of possible covariates in the analysis between iodine status and intelligence of the child, as measured by intelligence quotient, derived from earlier publications and discussion between authors. Nodes indicate exposure (green), outcome (blue), confounders (red), and background variables (grey). Arrows represent assumed causal directions

Relevant confounders were selected based on previous publications that have investigated the relationship between maternal iodine status and childhood intelligence at age 8 years in the same cohort [32, 33], on the basis of which we created a Directed Acyclic Graph (DAG) using *dagitty* software to select the minimal sufficient adjustment set (Fig. 2). The minimal sufficient adjustment set of confounders considers only confounders that influence both the exposure (maternal iodine status during the first trimester of gestation) and the outcome (intelligence of the child), which were: maternal education (low = no qualifications, certificate of secondary education or vocational training, medium = O levels, high = A level or degree), parity (0, 1, ≥ 2), maternal age (years), maternal pre-pregnancy BMI (kg/m^2^ calculated based on self-reported weight and height before pregnancy), ethnicity (White-British or other, if one or more parents of the mother were not White-British), and smoking during early pregnancy (ever smoking one or more cigarette daily in early pregnancy or never smoking). These variables were derived from questionnaires administered during pregnancy, i.e. before the exposure occurred. After careful consideration, ethnicity was not included as a covariate because the cohort was ethnically homogeneous (> 98% White-British). The very small number of participants of other ethnicities (< 2%) precluded reliable estimation and would have resulted in sparse categories and unstable model fits.

### Statistical analysis

We made use of R Studio version 4.3.2. and the packages 'rms', 'lmtest', and 'ggplot2'. First, we conducted a power analysis and imputed missing covariates, then we conducted analyses on the primary exposure, maternal iodine status, and the secondary exposure, maternal thyroid function (TSH and FT4), as described below.

#### Power analysis and missing analysis

Among participants whose intelligence was measured at age 15 years, valid iodine measurements were available for 1,211 mothers during early pregnancy. Based on a standard deviation of 15 IQ points and adjustment for eight predictors, this sample provides 80% power (α = 0.05) to detect a difference of approximately 3.3 IQ points. Covariates with less than 15% missing data were imputed using the 'missRanger' package in RStudio, which applies multiple imputation by chained equations with random forests over five iterations. All variables available at recruitment were included, with case weights accounting for both missingness and dropout since pregnancy.

#### Continuous analyses of the primary exposure

We examined the association between log-transformed urinary iodine status of the mother and intelligence outcomes (T-scores for Matrix Reasoning, T-scores for Vocabulary, and Two Subtests Full-Scale IQ) of their children at the age of 15 years, using regression models adjusted for confounders selected based on a directed acyclic graph (DAG; Fig. 2). To assess potential non-linear associations, we first compared three models: linear regression, quadratic regression, and restricted cubic splines (RCS) with three knots placed at the 10th, 50th, and 90th percentiles. Model selection was based on the Likelihood Ratio Test (LRT) and Akaike Information Criterion (AIC). Heteroscedasticity was assessed using the Breusch-Pagan test, which tests whether the variance of the residuals is constant. Adding a quadratic term or a restricted cubic spline did not improve model fit (data not shown). Based on this result, a linear regression model was chosen. To assess the robustness of our findings in the primary analysis, we repeated these after excluding mothers on thyroid hormone replacement therapy and excluding preterm (< 32 weeks) or very low birth weight (< 1500 g) infants. Next, we used logistic regression models to estimate odds ratios for being in the lowest tenth percentile of intelligence scores with maternal iodine status as predictor, both unadjusted and adjusted for aforementioned confounders. We also ran the continuous models with an interaction term for sex and maternal iodine status and sex-stratified for boys and girls separately when the interaction term was significant (*p* < 0.1).

#### Categorical analyses of the primary exposure

Next, we analysed the association between maternal iodine status and intelligence outcomes distinguishing between different cut-offs [32, 39]: < 50 µg/g: severely deficient, 50–100 µg/g: moderately deficient, 100–150 µg/g: mildly deficient, 150–250 µg/g: sufficient, > 250 µg/g: above adequate levels [32, 39]. In a regression analysis using the same confounders as in the continuous analyses, we entered these iodine status groups, as predictors with the sufficient iodine group (150–250 µg/g) as the reference group. We calculated Cohen's *d* to standardise effect sizes where comparisons were made between the three iodine deficiency groups and the sufficient iodine group as reference group, facilitating interpretation of the magnitude of the impact of iodine group on intelligence outcomes. Effect sizes were interpreted using conventional thresholds: 0.2 (small), 0.5 (medium), and 0.8 (large).

#### Iodine and maternal thyroid function in the first trimester

Next, our goal was to investigate whether any association was with maternal iodine status perse or due to the direct impact of her iodine status on her thyroid hormone supply and regulation of the thyroid axis. As the overlap between pregnancies with available urinary iodine and thyroid function measurement and intelligence outcomes at age 15 years was insufficient to achieve the same statistical power as the primary analysis, we instead conducted Pearson's correlation analysis between maternal iodine status and first-trimester thyroid function measurements (FT4 and TSH) in a subsample (n = 451) (Fig. 1) and repeated the linear regression models with first-trimester thyroid function measurements as predictor rather than iodine status. Additionally, we performed moderation analyses by rerunning all regression models selected in the primary analysis with an interaction term between iodine status and maternal thyroid function (i.e. separate models for iodine status*FT4 and iodine status*TSH), considering the full range of FT4 and TSH and the same confounders as in the primary analyses, stratifying by maternal iodine sufficiency based on the WHO cut-off of 150 µg/g if the interaction term between iodine status and thyroid function measurement was significant at *p* < 0.1 [32, 39].

## Results

We included 1,211 mother–child dyads with available maternal iodine status and intelligence outcomes at age 15 years. The median urinary iodine excretion adjusted for creatinine in the first trimester was 111.3 (µg/g) (IQR: 76.3–169.6), which is below iodine sufficiency according to the WHO cut-off, and the iodine status was on average determined at 9.16 ± 2.6 weeks (Table 1).

**Table 1:** Descriptive characteristics of the ALSPAC sample

Variable		Iodine measurement & WASI age 15 years (n = 1,211)	Subsample with iodine measurement and thyroid function available (n = 451)
Maternal age (Years)	Mean (SD)	29.6 (4.3)	29.3 (4.3)
Maternal education	% (Absolute number)	High: 47% (571)	High: 45% (201)
		Medium: 37% (442)	Medium: 39% (176)
		Low: 16% (198)	Low: 16% (74)
Pre-pregnancy BMI (kg/m2)	Median (IQR)	22.3 (20.7–24.5)	22.4 (20.9–24.4)
Ethnicity	% (Absolute number)	White-British: 98.8% (1197); Other: 1.2% (14)	White-British: > 99% (> 446)^a^; Other: < 1% (< 5)^a^
Parity	% (Absolute number)	0: 49% (597)	0: 47.89% (216)
		1: 33% (404)	1: 33% (152)
		≥ 2: 17% (210)	≥ 2: 18% (83)
Smoking in early pregnancy	% (Absolute number)	Never: 88% (1065)	Never: 88% (397);
		Yes: 12% (146)	Yes: 12% (54)
Iodine-to-creatinine ratio (µg/g)	Median (IQR)	111.3 (76.3–169.6)	105.9 (74.7–152.6)
Time of urinary iodine excretion testing (Gestational Weeks)	Mean (SD)	9.2 (2.6)	9.1 (2.5)
TSH (mIU/L)	Median (IQR)	–	0.89 (0.58–1.31)
FT4 (pmol/L)	Median (IQR)	–	16.15 (14.89–17.76)
Time of maternal thyroid function testing (weeks)	Mean (SD)	–	9.7 (2.1)
Sex assigned at birth	% (Absolute number)	Male: 47% (569)	Male: 50% (226)
		Female: 53% (642)	Female: 50% (225)
Age at neurocognitive testing (years)	Mean (SD)	15.4 (15.3–15.5)	15.4 (15.3–15.5)
T-scores matrix reasoning	Mean (SD)	47.0 (8.2)	46.7 (8.2)
T-scores vocabulary	Mean (SD)	45.7 (11.5)	45.8 (11.3)
Two subtest Full-scale IQ	Mean (SD)	94.5 (12.8)	94.4 (12.6)

^a^Counts below 5 are suppressed and shown as “ < 5” to protect participant confidentiality. These values may include zero. Percentages and counts in the same row are rounded or presented in ranges to prevent deduction of exact values

### Performance intelligence-matrix reasoning T-scores

We found no significant association between maternal iodine status in the first trimester and Matrix Reasoning T-scores at age 15 (β = 0.09 95% CI: −0.38 to 0.57; *p* = 0.69; Cohen's *d* = 0.01) in either the primary or the sensitivity analysis (Supplementary 2). The interaction term with sex was not significant (*p* = 0.72), therefore sex-specific analysis was not pursued. Higher maternal urinary iodine excretion in the first trimester did not change the odds of being within the lowest 10th percentile of Matrix Reasoning T-scores (β = 0.99; 95% CI: 0.81–1.20; *p* = 0.89). Children in the severely iodine deficient group did not have lower Matrix Reasoning T-scores at age 15 years compared to those in the iodine sufficient group (*p* = 0.95; Cohen's *d* = −0.01).

### Verbal intelligence-vocabulary T-scores

Maternal iodine status in the first trimester was significantly and positively associated with Vocabulary T-scores at age 15 (β = 0.73; 95% CI: 0.10–1.36; *p* = 0.024; Cohen's *d* = 0.07). This observation was replicated in sensitivity analyses excluding mothers with thyroid replacement medication in pregnancy and preterm born children (Supplementary 2). We additionally found that with higher maternal iodine levels in the first trimester, the odds of being in the lowest tenth percentile of Vocabulary T-scores decreased significantly (OR = 0.81; 95% CI: 0.66–0.99; *p* = 0.045). The interaction term between sex and iodine levels was not statistically significant (*p* = 0.80), indicating that there was no evidence of effect modification by sex. For the severely iodine deficient group (< 50 µg/g), as compared to the iodine sufficient group (150–250 µg/g), there was a 4.25 point lower Vocabulary T-scores at age 15 (95% CI: −7.10 to −1.40; *p* < 0.01), corresponding to a Cohen’s d of –0.40, reflecting a small-sized effect. Compared to the iodine sufficient group, there was no significant difference in Vocabulary T-scores for the moderate deficient group (β = −1.34; 95% CI: −3.06 to 0.39; *p* = 0.13; Cohen’s d = −0.12), Cohen’s *d* = 0.12, nor for the mildly deficient group (β = −1.14; 95% CI: −2.98 to 0.69; *p* = 0.22; Cohen’s *d* = −0.11).

### Full-scale intelligence

**Fig. 3:**
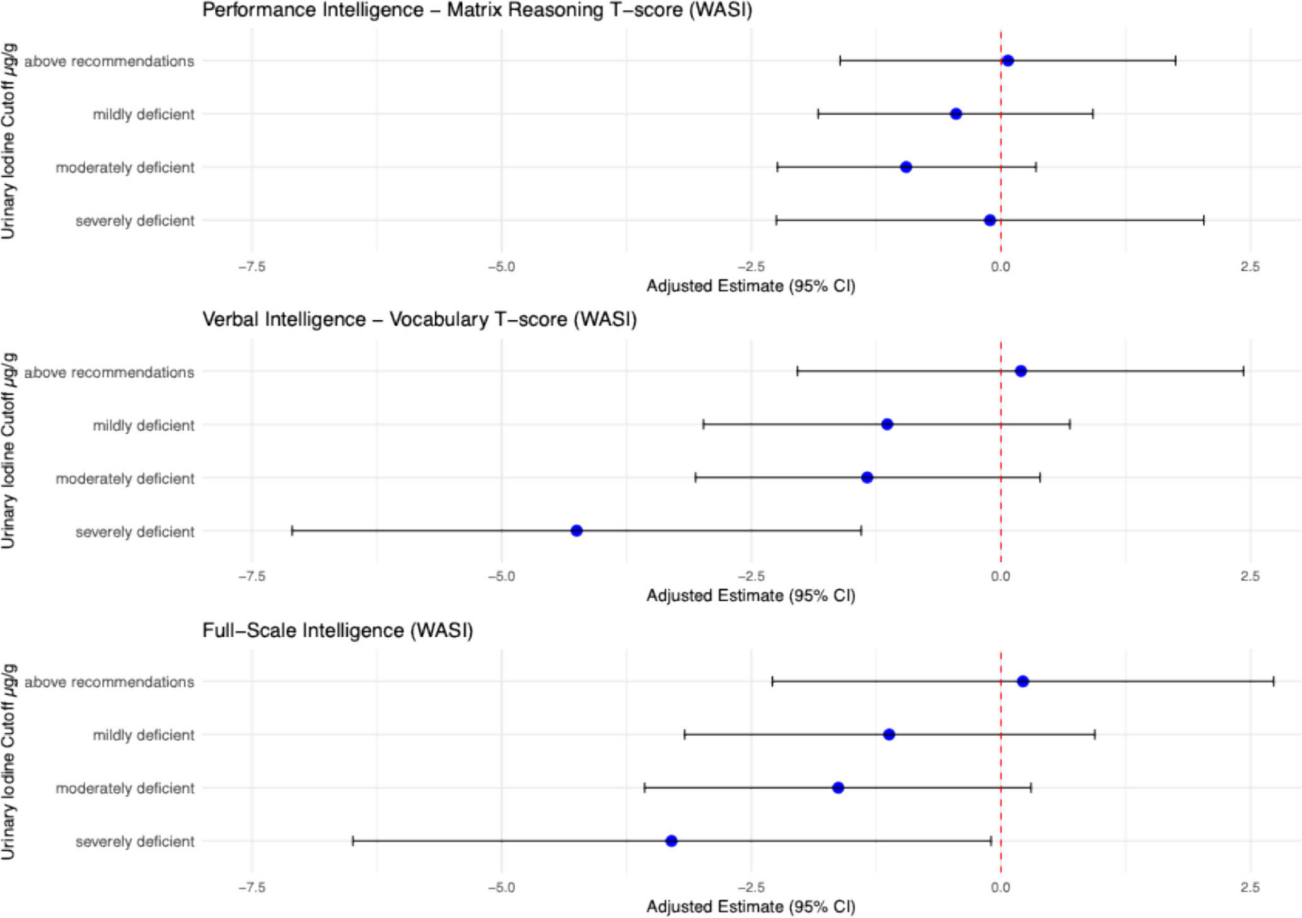
Forest plot of the intelligence score difference between the maternal iodine status categorised as severely iodine deficient (< 50 µg/g), moderately deficient (50–100 µg/g), mildly deficient (100–150 µg/g) or above recommendation group (> 250 µg/g), with iodine sufficiency (150–250 µg/g) as reference group. Estimates are adjusted for maternal education, parity, age, pre-pregnancy BMI, and smoking during early pregnancy

The association between maternal iodine status in the first trimester and Full-Scale Intelligence at the age of 15 was not significant (β = 0.60; 95% CI: −0.10 to 1.31; *p* = 0.094; Cohen's* d* = 0.05). After adjusting for confounders, the odds of being in the lowest tenth percentile of Full-Scale Intelligence were not decreased as iodine levels increased (OR: 0.88; 95% CI: 0.72 to 1.06; *p* = 0.19). The interaction term for sex was not significant (*p* = 0.97). However, children born from mothers severely deficient in iodine in the first trimester (< 50 µg/g) had lower IQ scores (β = –3.30; 95% CI –6.49 to –0.10; *p* = 0.04) compared to iodine sufficient pregnancies (150–250 µg/g), corresponding to a Cohen’s *d* of –0.27, indicating a small-sized effect (Fig. 3).

### Iodine and maternal thyroid function in the first trimester

Within this the subsample with both first-trimester maternal iodine status and thyroid function measurements available (n = 451), iodine concentration was not significantly correlated with FT4 (r = –0.04; 95% CI: –0.13 to 0.06; *p* = 0.43) or TSH (r = 0.01; 95% CI: –0.09 to 0.10; *p* = 0.88). Furthermore, neither FT4 nor TSH showed a significant association with intelligence outcomes after adjustment for the same confounders as in the primary analysis (Supplementary 3). We then explored whether the relationship between maternal thyroid function and child intelligence could be dependent on maternal iodine status. FT4 showed no meaningful differences across iodine status groups (all *p*-values > 0.66). For TSH, however, the association with child outcomes varied significantly by iodine status group (interaction *p*-values of 0.02 for Matrix Reasoning, 0.04 for Vocabulary, and < 0.01 for Full-Scale IQ). To explore this observation further, we divided the mother–child dyads in two groups using the WHO cut-off where < 150 µg/g is considered deficient and > 150 µg/g sufficient [51], and ran a linear regression model in both groups with TSH as predictor and the same confounders as in the primary analysis. In iodine-deficient pregnancies, higher maternal TSH was associated with higher child scores in Vocabulary and Full-Scale IQ at age 15 years (Table 2). In contrast, in the iodine-sufficient pregnancies, maternal TSH was not significantly associated with any of the children's intelligence outcomes. Matrix Reasoning showed no significant association with TSH in either group.

**Table 2:** Association between maternal TSH levels (log-transformed) and child intelligence outcomes stratified by maternal iodine status (> 150 µg/g as sufficient, < 150 µg/g as deficient)

	Deficient maternal iodine status in the first trimester (< 150 µg/g) (n = 334)				Sufficient maternal iodine status in the first trimester (> 150 µg/g) (n = 117)			
Outcome	Estimate	Confidence interval	*p*-value	Cohen's *d*	Estimate	Confidence interval	*p*-value	Cohen's *d*
Matrix reasoning T-score	0.36	−0.50 to 1.22	0.41	0.05	−1.26	−2.79 to 0.26	0.10	−0.16
Vocabulary T-score	1.29	0.17–2.42	0.02	0.13	−0.71	−2.80 to 1.38	0.50	−0.07
Full-scale IQ	1.4	0.17–2.63	0.03	0.12	−1.37	−3.72 to 0.98	0.25	−0.11

## Discussion

We assessed the association between maternal iodine status in early pregnancy and intelligence outcomes of adolescents at age 15 years in 1211 mother–child dyads from a prospective, population-based birth cohort study located in the former “goitre belt” in the UK. Our findings confirm that the positive association between iodine status during the first trimester and verbal intelligence outcomes observed at age 8 years persists into adolescence, irrespective of sex. Firstly, we found that iodine status during the first trimester was positively associated with Vocabulary T-scores at age 15 but not Matrix Reasoning or Full-Scale IQ. Using cut-offs for iodine deficiency we found that the children of mothers in the < 50 µg/g iodine group showed not only a significantly lower verbal intelligence but also a lower full-scale intelligence compared to the iodine sufficient reference group (150–250 µg/g), while adjusting for relevant socioeconomic confounders. This suggests that the observed association between iodine status and intelligence may be primarily driven by severe iodine deficiency rather than representing a continuous dose–response relationship. Children from mothers with higher maternal urinary iodine excretion in the first trimester had reduced odds of scoring in the lowest tenth percentile of Vocabulary T-scores at age 15. In the subsample with both iodine and thyroid function measurements available, we found no consistent associations between FT4 or TSH and adolescent intelligence outcomes but when investigating the interaction between thyroid function and intelligence, we found that the association between maternal thyroid function and intelligence is modified by iodine status. More specifically, an association between maternal TSH and intelligence outcomes was present in iodine-deficient pregnancies but not in iodine-sufficient pregnancies.

These findings are both novel and significant, as it suggests that the association between early iodine deficiency and verbal intelligence previously established in the ALSPAC cohort does not disappear over time but rather persists with age [32, 33]. Furthermore, the finding that maternal TSH predicts better intelligence outcomes in iodine-deficient but not sufficient pregnancies may reflect an adaptive mechanism by which elevated maternal TSH, within a compensated euthyroid state, enables sufficient FT4 production to support foetal neurodevelopment. This suggests that a flexible maternal thyroid axis may respond to iodine scarcity by upregulating TSH to maintain an euthyroid state, preserving foetal cognitive outcomes even under suboptimal nutritional conditions. As this hypothesis awaits further investigation, the current results underscore the importance of early public health interventions to prevent lasting cognitive deficits at the population level.

Our finding that children born from severely iodine deficient pregnancies have lower verbal and full-scale intelligence compared to the sufficient pregnancies, extends previous evidence that iodine status during early pregnancy affects offspring cognitive outcomes particularly in the verbal domain. It has previously been reported in the ALSPAC cohort that children of mothers with a urinary iodine-to-creatinine ratio (µg/g) below 150 µg/g at age 8 years were more likely to score in the lowest quartile for verbal IQ, reading accuracy, and comprehension, with the greatest deficits in the < 50 µg/g group [32]. In accordance with this earlier finding, we also found that the negative impact of maternal iodine status in the first trimester on adolescent intelligence is most pronounced in the verbal domain at age 15 years. Levie and colleagues [33] reported in an individual participant meta-analysis a positive curvilinear relationship between first-trimester maternal iodine status and verbal IQ but not non-verbal IQ. We similarly report at age 15 years an association with Vocabulary T-scores (verbal domain) but not Matrix Reasoning T-scores (non-verbal domain). A study from Italy further echoes these findings, observing a positive relationship between maternal iodine status in pregnancy and higher verbal intelligence in children aged 6–12 years [7]. Similar to these two studies, we found no direct correlation between maternal iodine status and maternal FT4 and TSH in the first trimester of pregnancy [7, 33]. This might be because thyroidal iodine storage plays an important role in buffering thyroid function from iodine deficiency; indeed, another study conducted in the iodine deficient context of the UK observed that lower preconception maternal iodine status (excretion < 50 µg/g) is associated with significantly lower child IQ at age 6–8 years, highlighting the importance of preconception iodine sufficiency for optimal neurodevelopment during early pregnancy [52]. However, as we additionally found that in iodine-deficient pregnancies, higher maternal TSH levels are related to better intelligence outcomes, this points towards a potential adaptive physiological compensation by the pregnant mother in face of iodine scarcity, a pattern which we did not observe in iodine sufficient pregnancies.

Although the difference in IQ scores between children of mothers with severe iodine deficiency (< 50 µg/g) and those with sufficient iodine levels (150–250 µg/g) was modest, approximately 3.3 IQ points (β = –3.30; Cohen’s *d* = –0.27), such small shifts can be meaningful at the population level. While a 3-point difference may not indicate clinical impairment in an individual child, it can increase the proportion of children falling below educational or developmental thresholds across a population [9]. Our findings suggest that intervention in maternal iodine status periconception may hold important population-level benefits in a mild-to-moderate iodine deficient region such as the UK. The implementation of iodine prophylaxis programmes in China since the 1990s, emphasise the substantial benefits of population-wide iodine supplementation in severely deficient areas, with gains in intelligence equating to approximately 8.7 IQ points when iodine-deficient communities are supplemented during pregnancy [53]. Timing is critical, as supplementation at 4–6 weeks of gestation has been found to be more effective for improving neurodevelopmental outcomes than later interventions when important early neurodevelopment has already taken place [54]. A systematic review of available randomised controlled trials on iodine supplementation in pregnancy has, however, emphasised that there is insufficient high-quality evidence to support iodine supplementation in areas of mild-to-moderate deficiency during pregnancy, possibly because these interventions occur too late in pregnancy, calling for robust randomised controlled trials that consider preconception iodine stores [18]. Together, these findings highlight that the safeguarding of iodine sufficiency in pregnancy should begin before conception and during early pregnancy, and the need for public health programmes to address iodine deficiency in reproductive-age women to prevent long-term cognitive deficits at a population level.

It is unclear what mechanism underlies the association between maternal iodine status and verbal intelligence, but not non-verbal intelligence, observed in our study as well as in other studies [7, 32, 33]. One possibility is that iodine deficiency in early pregnancy affects the development of the auditory system, particularly during the formation and maturation of the cochlea, which has sensitive windows of development for thyroid-hormone dependent processes [55, 56]. Enhanced access to iodine-rich shore-based diets during human evolution may have supported the energy-intensive processes driven by thyroid hormones, which are crucial for the development of the auditory system and the brain regions responsible for language acquisition, facilitating cognitive and linguistic advancements in humans [1, 57]. This hypothesis is supported by the observation that severe iodine deficiency, as seen in cretinism, causes congenital deafness amongst other developmental deficits [11, 55, 58]. However, the evidence for mild-to-moderate iodine deficiency impacting auditory development remains sparse [55].

This study has several limitations. Firstly, the incomplete overlap between maternal iodine measurements in the first trimester and thyroid function measurements during pregnancy in the ALSPAC cohort restricted our ability to determine whether maternal thyroid function, rather than iodine status itself, drives the observed associations. Yet for those individuals with both maternal thyroid function and iodine measurements in the first trimester available, we did not find a significant correlation between the two. However, we were not able to investigate other biomarkers of iodine deficiency or thyroid function in relationship to offspring neurodevelopment, which could clarify mechanisms underlying the associations. At age 15 years, only an abbreviated version of intelligence testing was conducted, which, while offering a reasonable estimate of IQ, lacks the precision of a full-scale intelligence assessment and, due to the lack of overlap in subtests with earlier assessments, prevented analysis of changes in IQ over time. Additionally, the subtests we use in our analysis will be indicative of the relevant domain, but have not been validated to predict either verbal or performance intelligence for clinical use. Furthermore, the predominantly White-British sample limits the generalisability of findings to populations with different ethnic backgrounds and dietary practices. While we adjusted for maternal education, which may be the underlying cause of differences in dietary diversity and iodine intake, this variable is a limited indicator of socioeconomic status. Additionally, we did not examine whether other nutritional deficiencies associated with reduced iodine intake might contribute to the observed associations, though Bath et al. [32] found no such effects at age 8 years. We were unable to assess the impact of iodine excess in this dataset, as it reflects a context of mild-to-moderate iodine deficiency and due to concern over contamination of the test strips in samples with high iodine levels.Further research is needed to confirm the curvilinear relationship observed by Levie et al. (2019) and explore the effects of iodine excess on neurodevelopmental outcomes. Finally, we did not examine the potential mediating influence of childhood iodine intake on found associations, which may also be affected postnatally by the mild-to-moderate iodine-deficient context of the UK. Prior research has however shown variability in iodine intake among UK children and adolescents, with younger children potentially protected from iodine deficiency by higher milk consumption, the primary dietary source of iodine in the UK, from which school-aged children derive about half of their daily iodine intake [30, 59, 60]. These findings highlight the need to understand how childhood iodine intake, influenced by diet and geography, may mediate or exacerbate the effects of iodine deficiency in early pregnancy.

In conclusion, this study shows that the association between iodine status in early pregnancy and children's intelligence, particularly in the verbal domain, persists into adolescence. These findings extend previous work to age 15 years in a mild-to-moderately deficient iodine context. Moreover, we find that iodine deficiency modifies the association between maternal thyroid function and intelligence outcomes. These results emphasise the lasting impact of early iodine deficiency in gestation on child neurodevelopment and the need for timely interventions [52]. Importantly, the associations were most pronounced in pregnancies severely deficient in iodine, supporting the case for a targeted approach. Future research should explore the relative roles of maternal and childhood iodine intake and elucidate mechanisms by which severe iodine deficiency may selectively impair verbal, but not performance, intelligence domains. National strategies to improve iodine status in women of reproductive age, especially those consuming diets low in iodine such as plant-based or dairy-free diets [5], are essential to optimise thyroidal iodine stores periconception and reduce the burden of iodine deficiency on children's neurodevelopment.

## Supplementary Information

Below is the link to the electronic supplementary material.

Supplementary file1 (DOCX 15 KB)

Supplementary file2 (DOCX 16 KB)

Supplementary file3 (DOCX 22 KB)

## Data Availability

Please note that the ALSPAC study website contains details of all the data that are available through a fully searchable data dictionary and variable search tool: http://www.bristol.ac.uk/alspac/researchers/our-data/. The study website contains information how to access the data by request to the ALSPAC Executive Committee.
