# Supplementary material for: Iodine deficiency in the first pregnancy trimester and intelligence in adolescence
Source: Eur J Nutr. 2026 Mar 31;65(3):111. doi: 10.1007/s00394-026-03955-3 (PMC13038719; doi:10.1007/s00394-026-03955-3)
Supplement: Supplementary file 1 — Supplementary file1 (DOCX 15 KB) [file 394_2026_3955_MOESM1_ESM.docx]

# Supplementary 1 Missing data report before imputation

| Variable | Missing Count (n=) | Missing Percentage (%) |
| --- | --- | --- |
| Maternal education | 88 | 4.69 |
| Maternal age | 0 | 0.00 |
| Smoking in early pregnancy | 24 | 1.28 |
| Parity | 41 | 2.18 |
| Ethnicity | 61 | 3.25 |
| Pre-pregnancy BMI | 104 | 5.54 |
