# Supplementary material for: Iodine deficiency in the first pregnancy trimester and intelligence in adolescence
Source: Eur J Nutr. 2026 Mar 31;65(3):111. doi: 10.1007/s00394-026-03955-3 (PMC13038719; doi:10.1007/s00394-026-03955-3)
Supplement: Supplementary file 2 — Supplementary file2 (DOCX 16 KB) [file 394_2026_3955_MOESM2_ESM.docx]

# Supplementary 2: Sensitivity analysis excluding preterm (<32 weeks) and low birth weight (<1500 g) babies and mothers taking thyroid hormone replacement therapy

| Outcome | Term^§^ | Unadjusted Estimate | Unadjusted SE | Unadjusted_p-value | Adjusted Estimate | Adjusted SE | Adjusted  p-value | CI Lower | CI Upper |
| --- | --- | --- | --- | --- | --- | --- | --- | --- | --- |
| T-Score Matrix Reasoning | Iod/Creat Ratio | 0.41 | 0.24 | 0.089 | 0.09 | 0.24 | 0.705 | -0.38 | 0.57 |
| T-Score Vocabulary | Iod/Creat Ratio | 1.26 | 0.33 | <0.01 | 0.74 | 0.32 | 0.021 | 0.11 | 1.38 |
| Full-Scale IQ | Iod/Creat Ratio | 1.27 | 0.37 | <0.01 | 0.62 | 0.36 | 0.088 | -0.09 | 1.33 |

§ Iod/Creat Ratio was log-transformed and scaled before analysis

Estimates are adjusted for the confounders maternal education, parity, age, pre-pregnancy BMI, and smoking during early pregnancy.
