# Supplementary material for: Iodine deficiency in the first pregnancy trimester and intelligence in adolescence
Source: Eur J Nutr. 2026 Mar 31;65(3):111. doi: 10.1007/s00394-026-03955-3 (PMC13038719; doi:10.1007/s00394-026-03955-3)
Supplement: Supplementary file 3 — Supplementary file3 (DOCX 22 KB) [file 394_2026_3955_MOESM3_ESM.docx]

# Supplementary 3 Maternal thyroid function in first trimester and intelligence outcomes at age 15.5 years for mother-child dyads where there is also a urinary iodine measurement

| **Variable** |  | **Subsample with Thyroid Function and Iodine Measurement available (n=451)** |
| --- | --- | --- |
| Maternal Age (Years) | Mean (SD) | 29.27 (4.32) |
| Maternal Education | % (Absolute Number) | high: 44.57% (201); low: 16.41% (74); medium: 39.02% (176) |
| Prepregnancy BMI (kg/m2) | Median (IQR) | 22.4 (20.87-24.4) |
| Ethnicity | % (Absolute Number) | Non-white: <1% (<5)^a^; White British: >99% (>446)^a^ |
| Parity | % (Absolute Number) | ≥2: 18.4% (83); 0: 47.89% (216); 1: 33.7% (152) |
| Smoking in early pregnancy | % (Absolute Number) | never: 88.03% (397); yes: 11.97% (54) |
| Creatinine-adjusted Urinary Iodine Excretion (µg/g) | Median (IQR) | 105.94 (74.67-152.63) |
| Time of Urinary Iodine Excretion Testing (Gestational Weeks) | Mean (SD) | 9.1 (2.52) |
| TSH (mIU/L) | Median (IQR) | 0.89 (0.58-1.31) |
| FT4 (pmol/L) | Median (IQR) | 16.15 (14.89-17.76) |
| Time of maternal thyroid function testing (weeks) | Mean (SD) | 9.67 (2.13) |
| Sex assigned at birth | % (Absolute Number) | 1: 50.11% (226); 2: 49.89% (225) |
| Age at Neurocognitive testing (years) | Median (IQR) | 15.37 (15.26-15.5) |
| T-score Matrix Reasoning | Mean (SD) | 46.67 (8.24) |
| T-score Vocabulary | Mean (SD) | 45.81 (11.33) |
| Two Subtest Full-Scale IQ | Mean (SD) | 94.36 (12.58) |

### ^a^: Counts below 5 are suppressed and shown as “<5” to protect participant confidentiality. These values may include zero. Percentages and counts in the same row are rounded or presented in ranges to prevent deduction of exact values.

When including all participants with iodine and thyroid function available (n=451)

| Outcome | Exposure^§^ | Unadjusted Estimate | Unadjusted Std. Error | Unadjusted p-value | Adjusted Estimate | Adjusted Std. Error | Adjusted p-value | Adjusted CI Lower | Adjusted CI Upper |
| --- | --- | --- | --- | --- | --- | --- | --- | --- | --- |
| Matrix Reasoning | FT4 | -0.366 | 0.388 | 0.346 | -0.357 | 0.379 | 0.346 | -1.097 | 0.387 |
|  | TSH | 0.055 | 0.389 | 0.887 | 0.037 | 0.381 | 0.923 | -0.726 | 0.786 |
| Vocabulary | FT4 | -0.758 | 0.533 | 0.156 | -0.704 | 0.497 | 0.157 | -1.679 | 0.272 |
|  | TSH | 1.061 | 0.532 | 0.047 | 0.823 | 0.499 | 0.100 | -0.167 | 1.803 |
| Total IQ | FT4 | -0.933 | 0.592 | 0.116 | -0.884 | 0.551 | 0.109 | -1.962 | 0.198 |
|  | TSH | 1.009 | 0.592 | 0.089 | 0.801 | 0.554 | 0.149 | -0.302 | 1.889 |

### § All FT4 and TSH values were scaled before analysis, TSH values were log-transformed. Estimates are adjusted for the confounders maternal education, parity, age, pre-pregnancy BMI, and smoking during early pregnancy.

When including participants with iodine and thyroid function available but excluding mothers with overt thyroid disease, taking thyroid medication, or children born with a birth weight <1500 g or before 32 weeks of gestation (n=445)

| Outcome | Exposure^§^ | Unadjusted Estimate | Unadjusted Std. Error | Unadjusted p-value | Adjusted Estimate | Adjusted Std. Error | Adjusted p-value | Adjusted CI Lower | Adjusted CI Upper |
| --- | --- | --- | --- | --- | --- | --- | --- | --- | --- |
| Matrix Reasoning | FT4 | -0.181 | 0.393 | 0.645 | -0.194 | 0.383 | 0.613 | -0.948 | 0.559 |
|  | TSH | -0.112 | 0.393 | 0.775 | -0.138 | 0.386 | 0.721 | -0.896 | 0.621 |
| Vocabulary | FT4 | -0.374 | 0.539 | 0.488 | -0.308 | 0.500 | 0.538 | -1.291 | 0.675 |
|  | TSH | 0.760 | 0.538 | 0.158 | 0.446 | 0.503 | 0.376 | -0.543 | 1.434 |
| Total IQ | FT4 | -0.484 | 0.599 | 0.419 | -0.442 | 0.555 | 0.426 | -1.533 | 0.649 |
|  | TSH | 0.649 | 0.598 | 0.279 | 0.377 | 0.559 | 0.500 | -0.721 | 1.475 |

### § All FT4 and TSH values were scaled before analysis, TSH values were log-transformed. Estimates are adjusted for the confounders maternal education, parity, age, pre-pregnancy BMI, and smoking during early pregnancy.
